# A rare case of extraluminally pedunculated gastrointestinal stromal tumor with postoperative metastasis to pancreas

**DOI:** 10.1093/jscr/rjab422

**Published:** 2021-09-27

**Authors:** Shoko Ogawa, Masayoshi Nishihara, Takaya Nakanishi, Tamaki Maeda, Seiichi Hirota

**Affiliations:** Department of Surgery, Okinawa Prefectural Hokubu Hospital, Nago, 905-0017, Okinawa, Japan; Department of Surgery, Okinawa Prefectural Hokubu Hospital, Nago, 905-0017, Okinawa, Japan; Department of Diagnostic Pathology, Okinawa Prefectural Hokubu Hospital, Nago, 905-0017, Okinawa, Japan; Department of Diagnostic Pathology, Moriguchi Keijinkai Hospital, Osaka, 570-0021, Japan; Department of Pathology, Hyogo College of Medicine, Hyogo, 663-8501, Japan

## Abstract

The gastrointestinal stromal tumor (GIST) is the most common type of sarcomatous tumor of the gastrointestinal tract. Many GISTs appear as submucosal tumors with intraluminal protrusion. GISTs with malignant features have a high risk of postoperative recurrence or metastasis, usually to the liver or peritoneum. We present a case of gastric GIST with double rarities: arising completely extraluminally with a pedicle and postoperative metastasis to the pancreas. A woman in her seventies diagnosed with a large extraluminal gastric GIST underwent complete removal of the tumor. Nine months later, a solitary metastatic tumor in the pancreas was detected. Imatinib controlled metastasis well for four years before the tumor became resistant. The patient then had a partial pancreatectomy with splenectomy. She is currently free from recurrence. We genetically analyzed the primary and metastatic GISTs and found known mutations related to poor prognosis and imatinib resistance.

## INTRODUCTION

A gastrointestinal stromal tumor (GIST) is the most common type of sarcomatous tumor of the GI tract. GISTs usually manifest as smooth, submucosal tumors enlarging intraluminally. The general treatment is surgical resection, and adjuvant therapy is recommended for cases categorized as high risk by major criteria. We describe a unique case of a huge pedunculated GIST of the stomach presenting as an extraluminal growth, followed by exceedingly rare postoperative pancreatic recurrence.

## CASE REPORT

**Figure 1:**
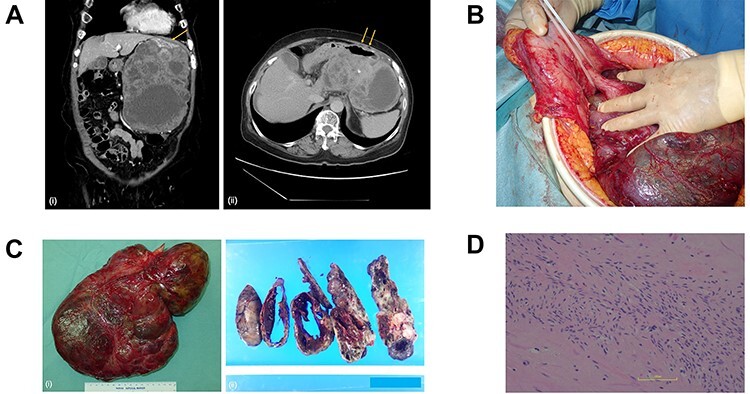
(**A**) Computed tomography scan of the abdomen before the primary surgery (i, coronal view; ii, axial view). The large pleomorphic mass measures 16×10×20 cm and is adjacent to the stomach (arrow). (**B**) Intraoperative inspection shows a large tumor pedunculated from the dorsal wall of the upper body of the stomach. (**C**) Postoperative specimen (i) of the gastric pedunculated GIST. The tumor shows (ii) pleomorphism of solid and cystic areas, calcification, hemorrhage, and necrosis. (**D**) The tumor consists mostly of spindle cells arranged in interlacing bundles and partially of epithelioid cells with severe atypia compared to usual GISTs.

A woman in her seventies was referred to our surgical unit, presenting with a history of pulsation on her abdomen and a left abdominal mass that she noticed one month earlier. She had no history of abdominal disorders or surgery. Computed tomography showed a huge mass (16 × 10 × 20 cm) arising extraluminally from the stomach (Fig. 1a).

The tumor was diagnosed as GIST by an immunohistochemical examination of the specimen from endoscopic ultrasound fine-needle aspiration. We started neoadjuvant chemotherapy using imatinib but discontinued it two months later due to febrile neutropenia. The patient underwent surgery, and intraoperative inspection showed a large tumor pedunculated from the dorsal wall of the upper body of the stomach (Fig. 1b). There was no visual evidence of metastasis or dissemination. The tumor was resected, including the pedicle and a small contiguous part of the stomach, without capsule rupture.

Grossly, the tumor (30 × 20 × 6 cm in size) proliferated from the gastric muscularis propria. The mass showed a mixture of solid and cystic areas, calcification, hemorrhage, and necrosis (Fig. 1c). Histologically the mitotic index was over 10 per 50 high-power fields (Fig. 1d). The final diagnosis was GIST, categorized as high risk of recurrence by all major prognostic criteria. The surgical margin was negative.

**Figure 2:**
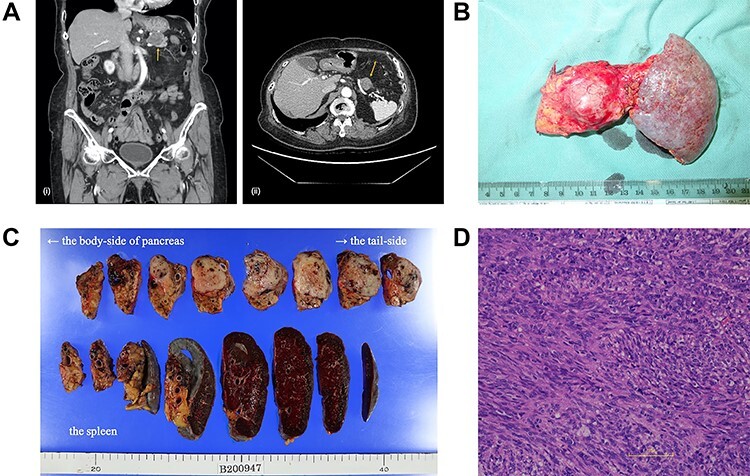
(**A**) A computed tomography scan of the abdomen was performed nine months after surgery. (i, coronal view; ii, axial view). A 2.2 cm mass has appeared at the anterior part of the tail of the pancreas (arrow). (**B**) The postoperative specimen including the recurrent tumor of pancreas from the dorsal side. (**C**) The tumor is solid without serosal invasion. The specimens on upper line are the pancreas, and those on lower line are the spleen. (**D**) The tumor cell structure shows more atypism than the original tumor.

The patient was discharged without complications, deciding against adjuvant chemotherapy despite being informed of the high risk of recurrence. Nine months later, a 2.2 cm mass appeared at the anterior part of the tail of the pancreas, adjacent to the gastric stump (Fig. 2a), and the patient chose chemotherapy in lieu of surgery. We started chemotherapy with low-dose imatinib (200 mg/day) because of the history of febrile neutropenia, subsequently lowering the dose to 100 mg/day due to side effects. She was able to continue the chemotherapy for four years without progression of metastasis.

Five years after the surgery, the mass had gradually expanded, indicating resistance to imatinib. This time, the patient elected to have surgery rather than the 2nd-line chemotherapy. The mass was attached to the splenic artery and vein, so we performed both distal pancreatectomy and splenectomy. The mass originated from the pancreas, not the stomach, with little adhesion to the gastric stump (Fig. 2b). No other metastatic lesion was observed.

The tumor in the specimen was 3.5 × 3.5 × 2.5 cm in size (Fig. 2c). The morphology and immunohistochemical staining showed features resembling those of the primary gastric GIST, although the tumor cells showed more atypism (Fig. 2d). The final diagnosis was GIST recurrence with a negative surgical margin. Now, six months after the second surgery, she is continuing low-dose imatinib therapy without recurrence.

We conducted a genetic alternation analysis of both the primary gastric GIST and the metastatic pancreatic tumor. Both tumors had the deletion of codons 557 and 558 of the c-kit exon 11, and the recurrent tumor had the additional substitution of Val with Ala in codon 654 (V654A) of the c-kit exon 13.

## DISCUSSION

In the two decades since the breakthrough of detecting c-kit mutations, many other genetic mutations of GISTs have been reported to be associated with various factors. In our case, the presence of c-kit mutations has an independent adverse influence on recurrence-free survival (RFS) [1]. In particular, deletion of the c-kit exon 11 557 and 558 seen in both tumors is associated with unfavorable RFS [1]. In contrast, the substitution of V654A in pancreatic metastasis is one of the most frequent mutations that result in resistance to imatinib [2]. In our case, these results suggest that the monoclonal proliferation of the tumor cells with V654A caused an acquired resistance to imatinib and subsequent tumor enlargement. This process is the general mechanism of GISTs developing resistance to imatinib. There was no other genetic mutations suggesting the relation to the two characteristics of our case: metastasis to the pancreas and extraluminal growth with a peduncule.

Pancreatic metastasis of GIST is extremely rare. The common metastatic sites of GIST are the liver and peritoneum, and they occur much less frequently in the lymph nodes, bones, and lungs [3]. A literature review revealed only three reported cases of GISTs metastasizing in the pancreas [4, 5]. In those cases, the pathologic details, including risk factors and genetic mutations, were not clarified. In our case, pathological results showed a negative surgical margin and no serosal invasion. As a result, hematogenous and/or lymphogenous metastasis could be suspected, although there was no pathological evidence.

We summarize our case and the additional 12 reported cases of adult extraluminally pedunculated gastric GISTs in Table 1 [6–14]. These tumors tended to be large (the median size was 12 cm), but they could be removed by simple wedge resection of the gastric wall around the peduncle, and they rarely recurred.

**Table 1:** Cases of Pedunculated Gastrointestinal Stromal Tumors

No.	Year	Age/Sex	Complaint	Preoperative diagnosis	Tumor size (cm)	Location	Rupture	Operative procedure	Mitotic rate (HPF)	Risk classification	Outcome (month)	NAC/AC	Recurrence	Author
1	2003	59/M	None	ND	9	ND	No	Wedge resection	ND	Not known	ND	ND	ND	Naitoh	
2	2004	84/F	None	ND	18.5	Posterior wall	No	Wedge resection	ND	High	Alive (ND)	None	No	Kimura	
3	2008	72/M	Abdominal pain, weight loss	ND	15	Larger gastric curvature	No	Partial gasterectomy	10+15/50	High	Alive (12)	None	No	Cavallaro	
4	2008	63/M	None	ND	19	Posterior wall	No	Partial gasterectomy	18/50	High	Alive (18)	None	No	Cavallaro	
5	2012	51/M	Abdominal pain	ND	6	Posterior wall	Yes	Tumorectomy	2/50	High	Alive (8)	None	No	Yakan	
6	2013	38/F	Abdominal pain, swelling, nausea, vomiting, weakness, loss of appetite	pancreas cancer	14	Great curvature	No	Tumorectomy	1/50	High	Alive (8)	AC	No	Baskiran	
7	2013	69/M	None	GIST	4	Front wall of antrum	No	Wedge resection	2/50	Low	Alive (18)	None	No	Parisi	
8	2017	74/F	Abdominal pain	ND	15	Posterior wall	No	Partial gasterectomy + splenectomy	10/50	High	Alive (20)	None	No	Wang	
9	2018	55/M	Abdominal pain, regurgation, heart burn	GIST	10	Antrum	No	Wedge resection	6/50	High	Alive (ND)	AC	No	Alkaaki	
10	2020	62/M	None	GIST	10	Antrum	No	Tumorectomy	≦5/50	Intermediate	Alive (12)	None	No	Wang	
11	2020	63/F	Abdominal pain, non bloody emesis, nausea, dark stool	ND	7	Great curvature	Yes	Partial gasterectomy	24/50	High	Alive (ND)	AC	No	Shively	
12	2020	79/F	Abdominal pulsation and mass	GIST	30	Gastric body	No	Wedge resection	≧10/50	High	Alive (6)	NAC + AC	Yes	Our case	

AC, adjuvant chemotherapy; HPF, high-power fields; NAC, neoadujuvant chemotherapy; ND, not described.

Retrospectively reflecting on our case, we recognize some potential improvements. First, if we had been aware of the mutation with a poor prognosis soon after the initial operation, we could have suggested adjuvant therapy more strongly. An early administration of imatinib might have better-controlled recurrence. Second, we could have scheduled the surgery earlier before the recurrent tumor became resistant to imatinib, resulting in a better prognosis [15]. The median time for GISTs to acquire imatinib resistance is two years, and surgery before the resistance results in a better prognosis [15].

In conclusion, this rare type of GIST has encouraged us to consider analyzing genetic alternations more often.

## CONFLICT OF INTEREST STATEMENT

None declared.

## FUNDING

None.

## References

[1] Zhang H, Liu Q. Prognostic indicators for gastrointestinal stromal tumors: A review. Transl Oncol 2020;13:100812. 32619820 10.1016/j.tranon.2020.100812 PMC7327422

[2] Robert GK, Odell FA, Byrnes ME, Baleato MR, Griffith R, Lyons BA, et al. Resistance to c-KIT kinase inhibitors conferred by V654A mutation. Mol Cancer Ther 2007;6:1159–66. 17363509 10.1158/1535-7163.MCT-06-0641

[3] DeMatteo RP, Lewis JJ, Leung D, Mudan SS, Woodruff JM, Brennan MF. Two Hundred Gastrointestinal Stromal Tumors, Recurrence Patterns and Prognostic Factors for Survival. Ann Surg 2000;231:51–8. 10636102 10.1097/00000658-200001000-00008 PMC1420965

[4] Geramizadeh B, Kashkooe A, Nikeghbalian S, Malek-Hosseini SA. Metastatic Tumors to the Pancreas, a Single Center Study. Arch Iran Med 2019;22:50–2. 30821161

[5] Reddy S, Wolfgang CL. The role of surgery in the management of isolated metastases to the pancreas. Lancet Oncol 2009;10:287–93. 19261257 10.1016/S1470-2045(09)70065-8

[6] Naito I, Okayama Y, Hirai M, Kitajima Y, Hayashi K, Okamoto T, et al. Exophytic pedunculated gastrointestinal stromal tumor with remarkable cystic change. J Gastroenterol 2003;38:1181–4. 14714258 10.1007/s00535-003-1228-2

[7] Kimura H, Yoshida T, Kinoshita S, Takahashi I. Pedunculated giant gastrointestinal stromal tumor of the stomach showing extragastric growth: report of a case. Surg Today 2004;34:159–62. 14745619 10.1007/s00595-003-2659-3

[8] Cavallaro G, Sadighi A, Polistena A, Rossi V, Cristaldi M, et al. Pedunculated giant GISTs of the stomach with exophytic growth: report of two cases. Int J Surg 2008;6:80–2. 10.1016/j.ijsu.2007.04.002 17513182

[9] Yakan S, Ilhan E, Cengiz F, Mollamehmetoglu H, Telciler KM. Acute abdomen caused by nontraumatic hemoperitoneum is the first manifestation of gastric low grade stromal tumor. World J Emerg Med 2012;3:232–4. 25215069 10.5847/wjem.j.issn.1920-8642.2012.03.013 PMC4129777

[10] Parisi A, Desiderio J, Trastulli S, Pressi E, Minicucci A, Farinacci F, et al. Laparoscopic single-stapling gastric transection for exophytic pedunculated gastrointestinal stromal tumor : is a safe procedure ? Surg Laparosc Endosc Percutan Tech 2013;23:e93–7. 23752027 10.1097/SLE.0b013e3182773f3c

[11] Wang L, Liu L, Liu Z, Tian Y, Lin Z. Giant gastrointestinal stromal tumor with predominantly cystic changes: a case report and literature review. World J Surg Oncol 2017;15:220. 29237476 10.1186/s12957-017-1285-2 PMC5729273

[12] Alkaaki A, Abdulhadi B, Aljiffry M, Nassif M, Al-Maghrabi H, Maghrabi AA. Coexistence of primary GEJ adenocarcinoma and pedunculated gastric gastrointestinal stromal tumor. Case Rep Surg 2018;2018:43788368. 10.1155/2018/4378368 PMC6016216 29992077

[13] Wang M, Qiu X, He X, Tian C. Characteristic of extra luminal gastric stromal tumor arising from the lesser curvature of the stomach. Medicine. 2020;99:16(e19885) 32312014 10.1097/MD.0000000000019885 PMC7220736

[14] Shively J, Ebersbacher C, Walsh WT, Allemang MT. Spontaneous hemoperitoneum from a ruptured gastrointestinal stromal tumor. Cureus 2020;12:e9338. 32850212 10.7759/cureus.9338 PMC7444847

[15] Raut CP, Posner M, Desai J, Morgan JA, George S, Zahrieh D, et al. Surgical management of advanced gastrointestinal stromal tumors after treatment with targeted therapy using kinase inhibitors. J Clin Oncol 2008;24:2325–31. 10.1200/JCO.2005.05.3439 16710031

